# Supplementary material for: Area-selective atomic layer deposition on 2D monolayer lateral superlattices
Source: Nat Commun. 2024 Mar 8;15:2138. doi: 10.1038/s41467-024-46293-w (PMC10924103; doi:10.1038/s41467-024-46293-w)
Supplement: Supplementary file 1 — Supplementary Information [file 41467_2024_46293_MOESM1_ESM.pdf]

Area-selective atomic layer deposition on 2D monolayer lateral superlattices

Supplementary Information

I. Supplementary Figures

Fig. 1 Additional characterizations of monolayer MoS_2-$MoSe_2$ lateral superlattice
Fig. 2 Various materials AS-ALD on the monolayer MoS_2-$MoSe_2$ lateral superlattice
Fig. 3 Additional characterizations of Al_2O_3 SAS-ALD
Fig. 4 Al_2O_3 SAS-ALD on MoS_2-$MoSe_2$ lateral superlattice with large $MoSe_2$ width
Fig. 5 Additional MD simulation mapping
Fig. 6 Schematic of dissociative adsorption of TMA on TMD
Fig. 7 Charge density difference plots of TMA on TMD surfaces for chemisorption selectivity
Fig. 8 Selectivity loss in SAS-ALD by multiple vacancy defects
Fig. 9 TMA adsorption energy under separate x and y lattice straining
Fig. 10 TMA adsorption on concave surface of TMD
Fig. 11 Adsorption selectivity of H_2O in SAS-ALD
Fig. 12 Adsorption energy of ligand in ALD precursors for Al_2O_3, Sb_2Se_3, HfO_2, and Te
Fig. 13 Adsorption energy of other ALD precursors
Fig. 14 Gibbs free energy for adsorption of TMA on 2D surface and the diffusion barriers
Fig. 15 Al_2O_3 ALD on single MoS_2 and $MoSe_2$ surface
Fig. 16 Diffusion and desorption rate varying with temperature
Fig. 17 TMA equilibrium coverage obtained by kMC
Fig. 18 Heatmap of TMA collisions with varying temperature
Fig. 19 Al_2O_3 SAS-ALD on WS_2-WSe_2 lateral superlattice
Fig. 20 Interface quality and optical property of selectively deposited aluminum oxide
Fig. 21 Electrical characterization of $MoSe_2$ nanoribbon FET (NRFET)

II. Supplementary Table

Table. 1 Adsorption and reaction energies on TMD surface sites

III. Supplementary Notes

Supplementary Note 1 Descriptions of CAS-ALD researches
Supplementary Note 2 Characterization of MoS_2, $MoSe_2$ regions in lateral superlattice
Supplementary Note 3 Periodic deposition of Al_2O_3 on $MoSe_2$ ripple and buckle
Supplementary Note 4 MD simulation strain mapping in triangular cell
Supplementary Note 5 Reaction and defect effects in SAS-ALD
Supplementary Note 6 The adsorption energy trend for various precursors and temperature
Supplementary Note 7 Additional information on kinetic Monte Carlo simulation
Supplementary Note 8 Interface and optical property of selectively deposited aluminum oxide
Supplementary Note 9 Fabrication of $MoSe_2$ nanoribbon FET (NRFET)

IV. Supplementary References

I. Supplementary Figures

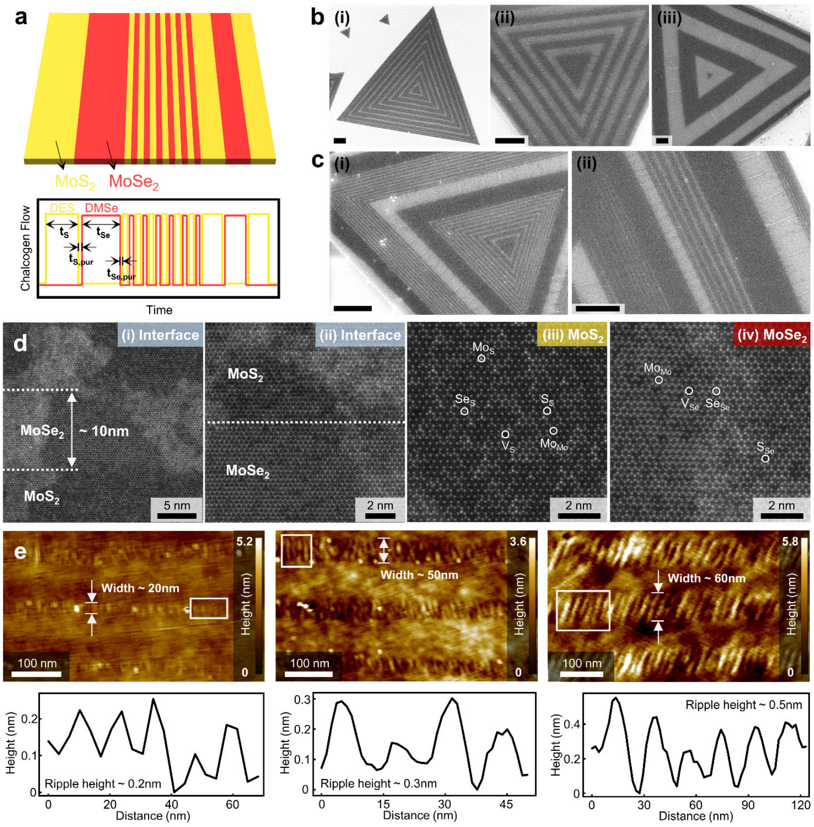

Supplementary Fig. 1. Additional characterizations of monolayer MoS_2-$MoSe_2$ lateral superlattice. a Schematic of lateral superlattice growth with various widths. The width is controlled by the injection time of the gas phase precursor (diethyl sulfide and dimethyl selenide). **b** SEM images of lateral superlattice with constant pitch size. The MoS_2/$MoSe_2$ width ratios are (i) 150 nm/50 nm, (ii) 115 nm/135 nm, and (iii) 640 nm/640 nm. **c** SEM images of lateral superlattice with various pitch size in a flake. Scale bar for (**b**) and (**c**), 500 nm. **d** HAADF-STEM images of MoS_2-$MoSe_2$ interface (i, ii), MoS_2 (iii), and $MoSe_2$ (iv) region in the lateral superlattice. **e** AFM images of MoS_2-$MoSe_2$ lateral superlattices with 20, 50, 60 nm $MoSe_2$ width.

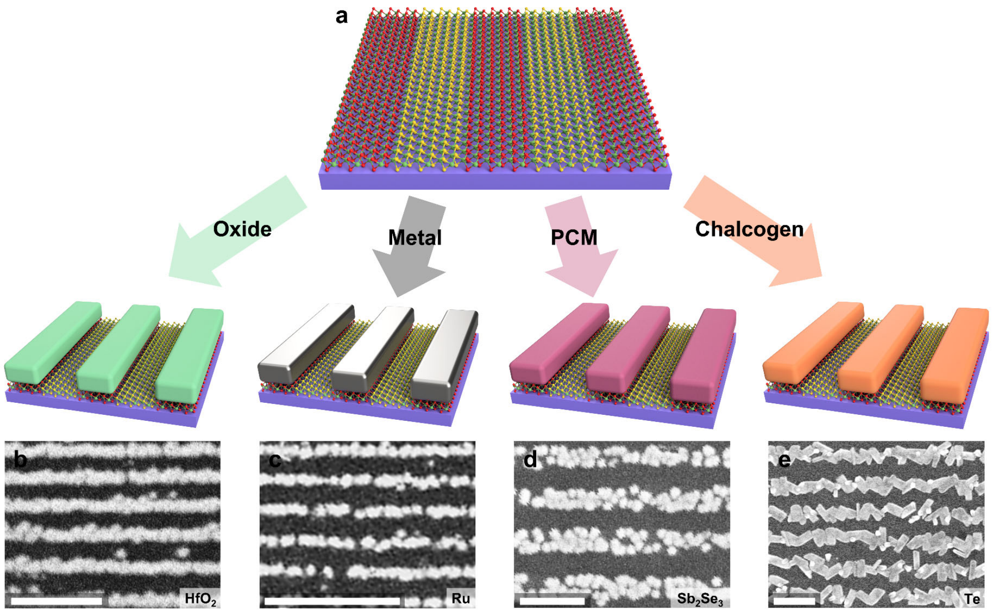

Supplementary Fig. 2. Various materials AS-ALD on the monolayer MoS_2-$MoSe_2$ lateral superlattice. a Schematics of oxide, metal, phase change material (PCM), and chalcogen AS-ALD. **b-e** SEM images of AS-ALD; HfO_2 (**b**), Ru (**c**), Sb_2Se_3 (**d**), and Te (**e**). Scale bar for (**b**)-(**d**), 300 nm.

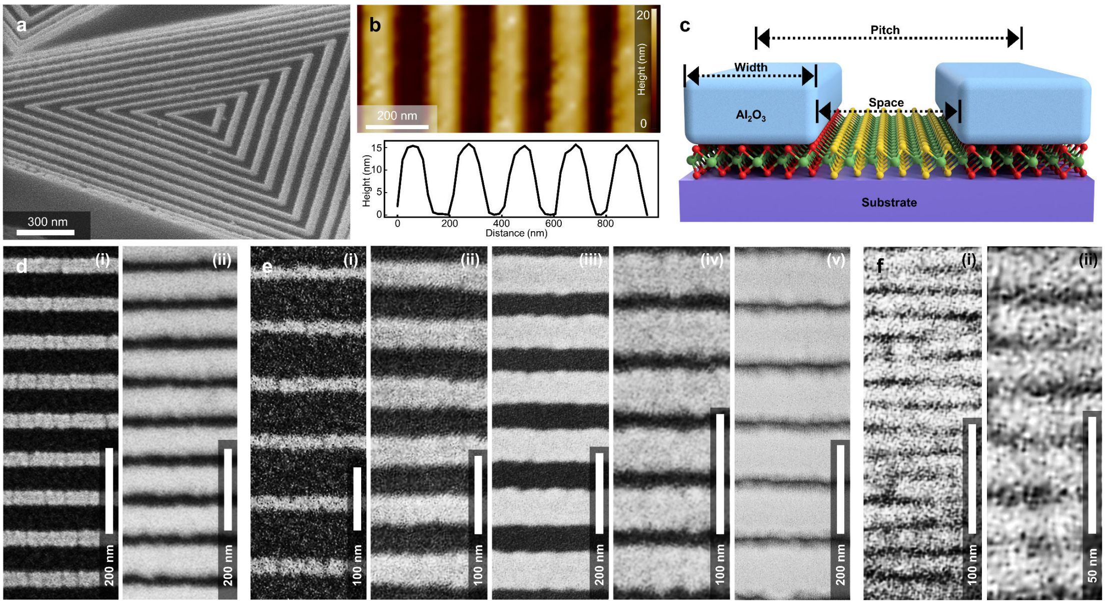

Supplementary Fig. 3. Additional characterizations of Al_2O_3 SAS-ALD. a Tilted SEM images after Al_2O_3 AS-ALD on the monolayer lateral superlattice. **b** AFM image of 15 nm AS-ALD structure. **c** Schematic for definition of width, space, and pitch size in the SAS-ALD structure. **d-f** SEM images of SAS-ALD structure representing same pitch size with reversed width-space ratio (**d**), control of width-space ratio (**e**), and minimum pitch size of SAS-ALD (**f**).

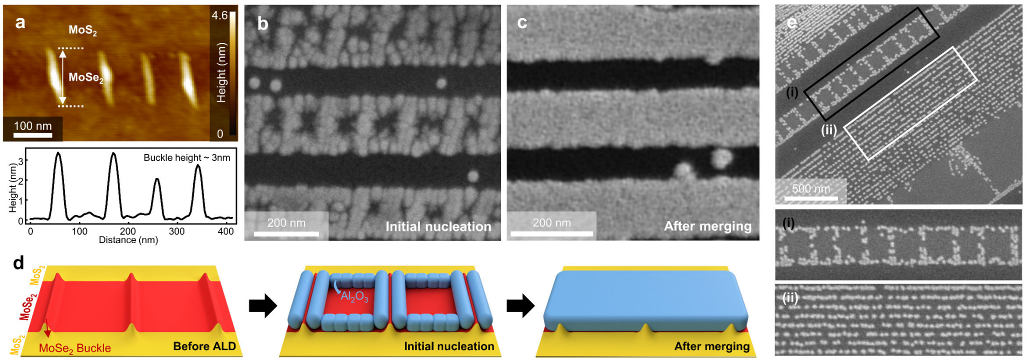

Supplementary Fig. 4. Al_2O_3 SAS-ALD on MoS_2-$MoSe_2$ lateral superlattice with large $MoSe_2$ width. a AFM image and height profile of the lateral superlattice which has large $MoSe_2$ width (> 100 nm). **b, c** SEM images of initial nucleation step (**b**, 20 ALD cycles), and after merging step (**c**, 100 ALD cycles). **d** Schematics for SAS-ALD process in large $MoSe_2$ width case. **e** SEM images of initial nucleation step (10 ALD cycles) in various width case (large $MoSe_2$ width in (i), small $MoSe_2$ width in (ii)).

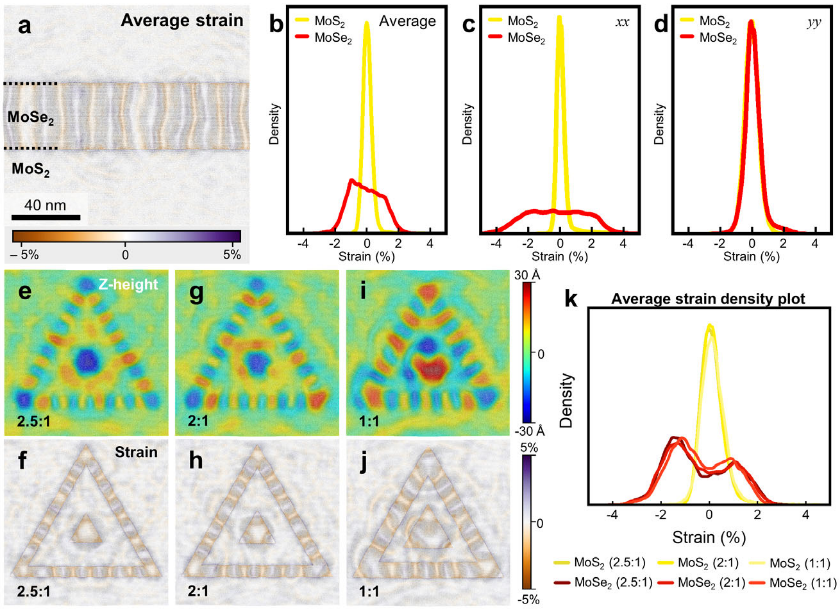

Supplementary Fig. 5. Additional MD simulation mapping. a Average strain mapping images of the lateral superlattice. **b-d** Strain density plot of average, *xx*, and *yy* (**Fig. 3d**) strain tensors. **e-j** *Z*-height and strain mapping data of triangular cell by MD simulation. The MoS_2:$MoSe_2$ width ratio is 2.5:1 (**e, f**), 2:1 (**g, h**), and 1:1 (**i, j**). **k** Average strain density plot of **e-j**.

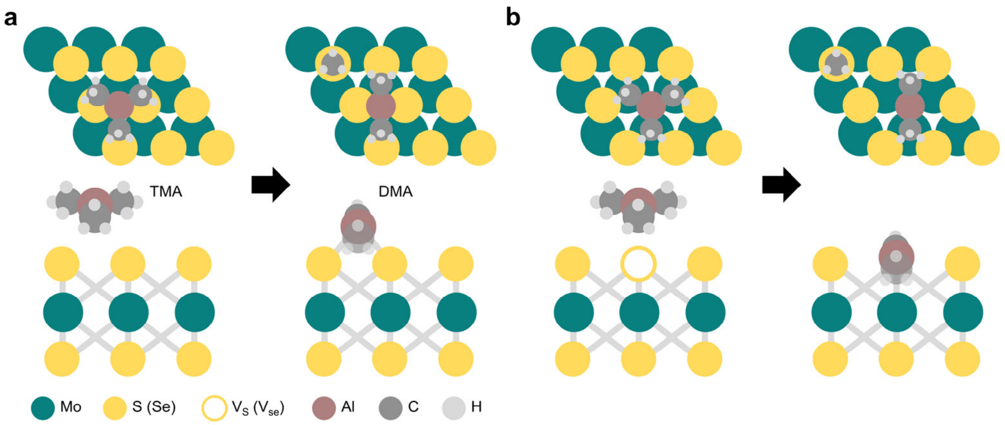

Supplementary Fig. 6. Schematic of dissociative adsorption of TMA on TMD. a Schematics of reaction between TMA and MoS_2 ($MoSe_2$) basal plane. The dimethyl aluminum (DMA) is bonded to the sulfur (selenium) bridge site. **b** Schematics of the reaction of TMA on a single sulfur (selenium) vacancy site.

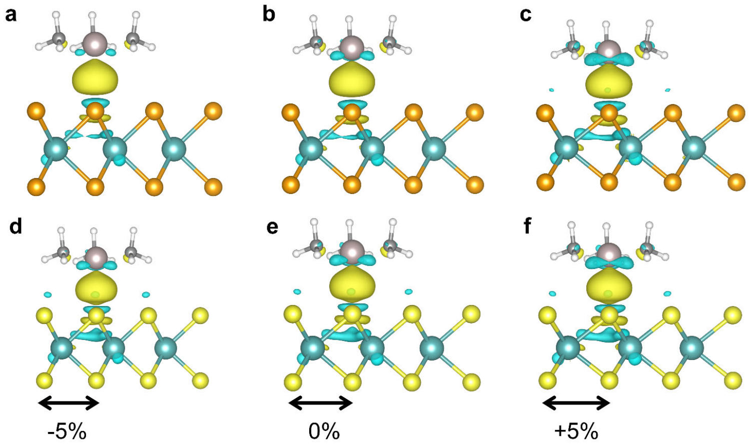

Supplementary Fig. 7. Charge density difference plots of TMA on TMD surfaces for chemisorption selectivity. a-f Charge density difference plots of $MoSe_2$ (**a-c**) and MoS_2 (**d-f**) with varying *x*, *y* lattice sizes of -5, 0, and +5 % (left to right). Iso-surface value of 0.001 (e/bohr^3) was used throughout the figures, with yellow representing electron depletion and cyan electron accumulation.

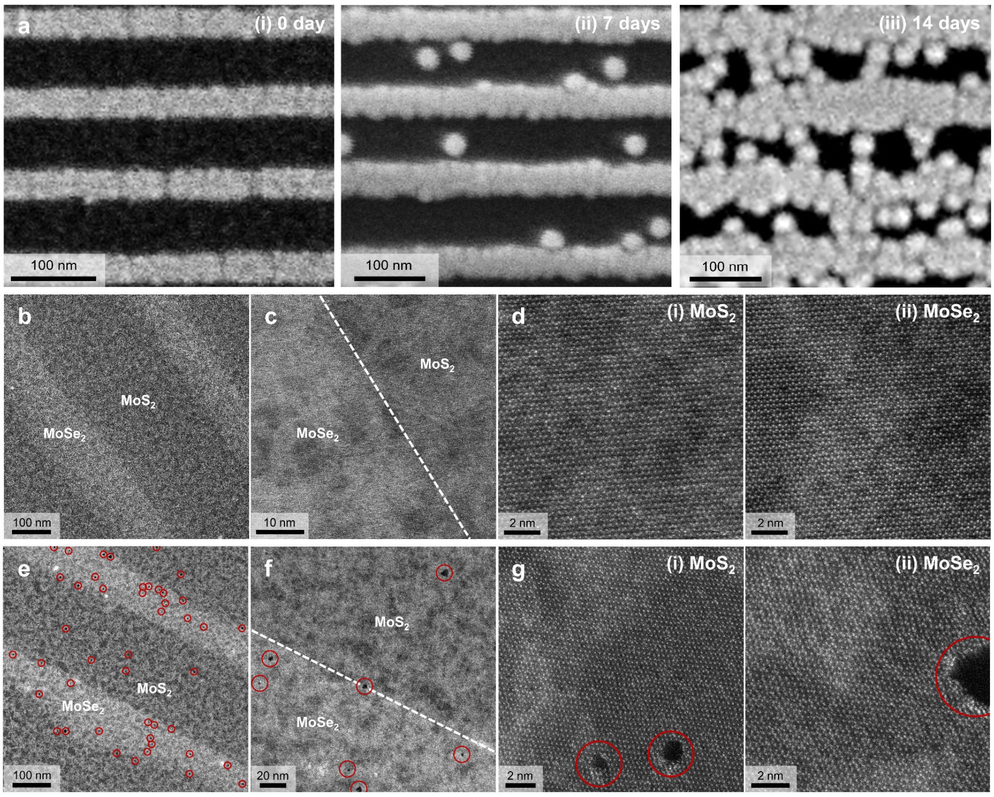

Supplementary Fig. 8. Selectivity loss in SAS-ALD by multiple vacancy defects. a-f SEM images of Al_2O_3 AS-ALD on lateral superlattices, which are non-degradation (i), 7 days degradation (ii), and 14 days degradation (iii) in air condition. **b-d** HAADF-STEM images of MoS_2-$MoSe_2$ lateral superlattice without degradation in air. There are no multiple vacancy defects in low magnification image (**b**), interface region (**c**), and MoS_2 and $MoSe_2$ regions (**d**). **e-g** HAADF-STEM images of the lateral superlattice with degradation in air for 14 days. Red circles in (**e-g**) represent multiple vacancy defects.

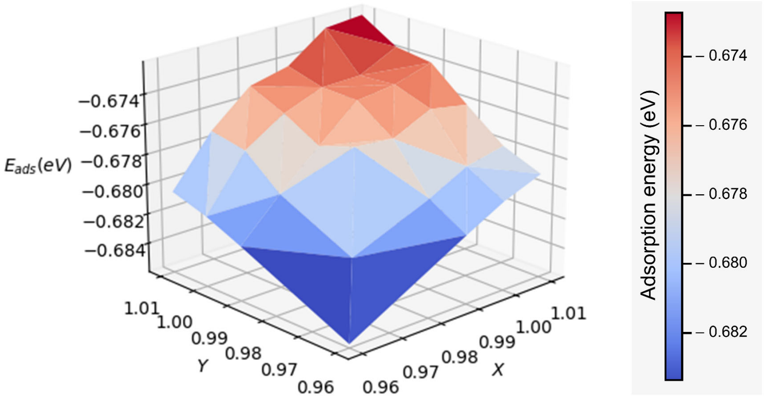

Supplementary Fig. 9. TMA adsorption energy under separate *x* and *y* lattice straining. The 3D graph shows the TMA adsorption energy when adjusting the *x* and *y* direction strain component separately.

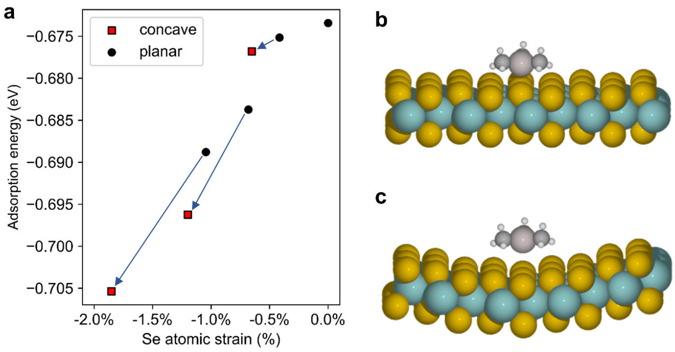

Supplementary Fig. 10. TMA adsorption on concave surface of TMD. a TMA adsorption energy against the *xx*, *yy*, *zz* average atomic strain of Se site on flat and curved $MoSe_2$ surface with varying compressive strain. **b, c** Schematics of TMA adsorption on flat (**b**) and concave (**c**) surface.

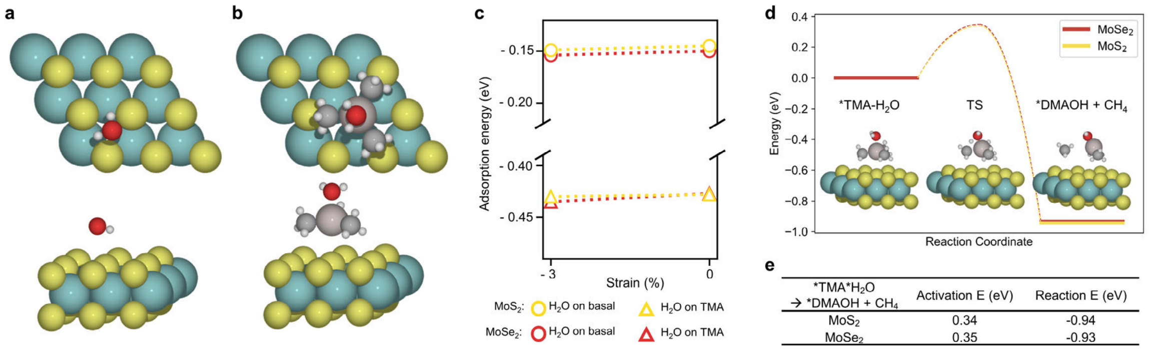

*TMA*H$_2$O → *DMAOH + CH$_4$	Activation E (eV)	Reaction E (eV)
MoS$_2$	0.34	-0.94
MoSe$_2$	0.35	-0.93

Supplementary Fig. 11. Adsorption selectivity of H_2O in SAS-ALD. a, b Schematics for adsorption of H_2O on MoS_2 basal plane (**a**) and pre-adsorbed TMA (**b**). **c** Adsorption energies of H_2O on basal plane and on pre-adsorbed TMA varying with strain. **d** Energy diagram for reaction of TMA-H_2O complex on MoS_2 and $MoSe_2$ surface. Insets are initial (*TMA-H_2O), transition (TS), and final states (*DMAOH+CH_4) of the complexes on TMD surface. **e** Activation energy and reaction energy of the reaction in **d**.

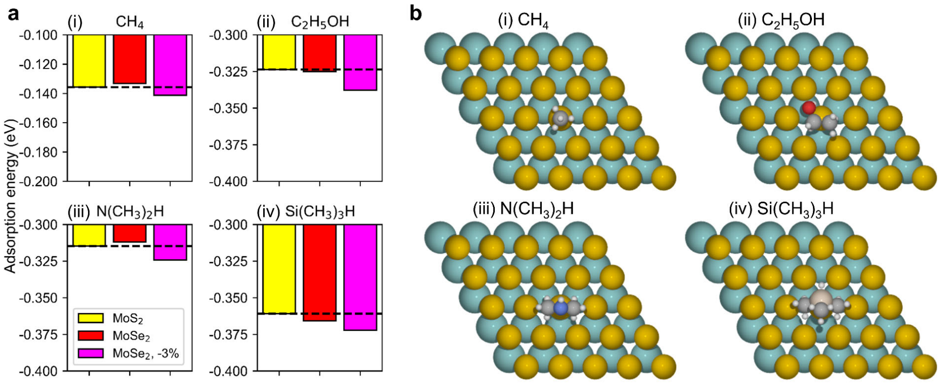

Supplementary Fig. 12. Adsorption energy of ligand in ALD precursors for Al_2O_3, Sb_2Se_3, HfO_2, and Te. a Adsorption energies of hydrogenated ligands used in various SAS-ALD precursors (i - Al_2O_3, ii - Sb_2Se_3, iii - HfO_2, iv - Te) measured on MoS_2, $MoSe_2$, and 3 % compressed $MoSe_2$. **b** Schematics of corresponding optimized structure for the ligands.

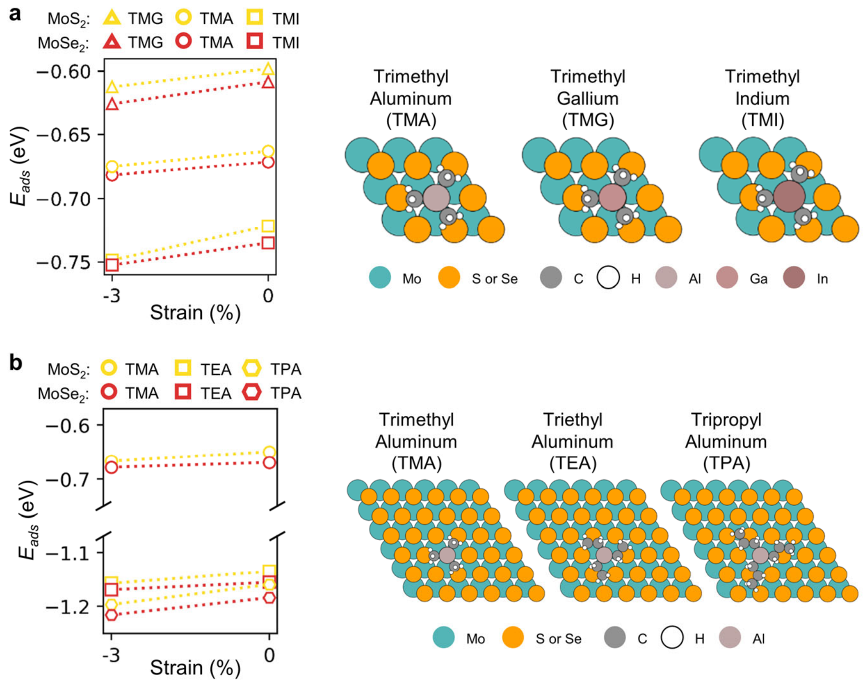

Supplementary Fig. 13. Adsorption energy of other ALD precursors. a, b Comparison of adsorption energy with varying central metals in precursors (**a**, TMA, TMG, TMI) and ligands in precursors (**b**, TMA, TEA, TPA).

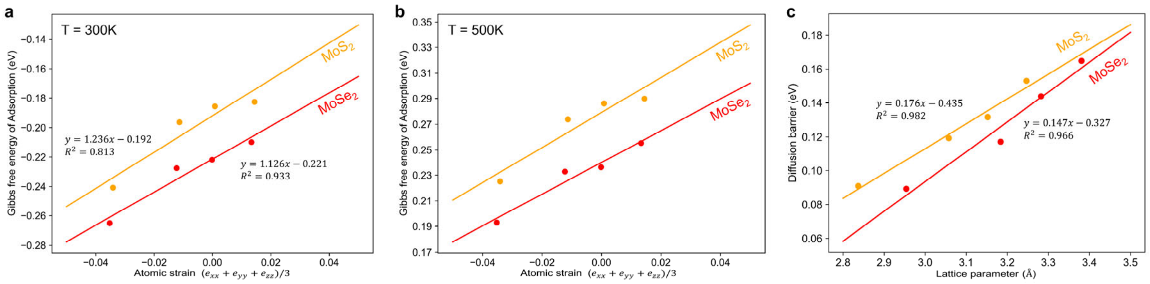

Supplementary Fig. 14. Gibbs free energy for adsorption of TMA on 2D surface and the diffusion barriers. a, b Gibbs free energy plot of TMA adsorption on MoS_2 (yellow) and $MoSe_2$ (red) at T = 300 K (**a**) and T = 500 K. (**b**) with varying atomic strain. The inset equations indicate the linear fit used to estimate the desorption rates. **c** Diffusion barriers on MoS_2 and $MoSe_2$ with varying lattice spacing, and its linear fit used to estimate the diffusion rates.

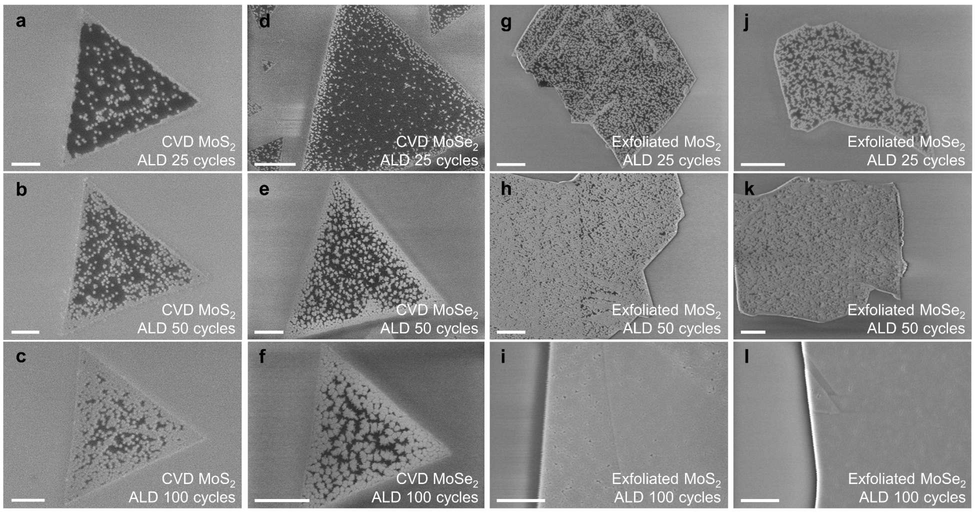

Supplementary Fig. 15. Al_2O_3 ALD on single MoS_2 and $MoSe_2$ surface. SEM images of Al_2O_3 ALD on CVD grown MoS_2 flake (**a-c**), CVD grown $MoSe_2$ flake (**d-f**), exfoliated MoS_2 flake (**g-i**), and exfoliated $MoSe_2$ flake (**j-l**). The cycles of ALD are 25 (**a, d, g, j**), 50 (**b, e, h, k**), and 100 (**c, f, i, l**). Scale bar for (**a-l**), 500 nm.

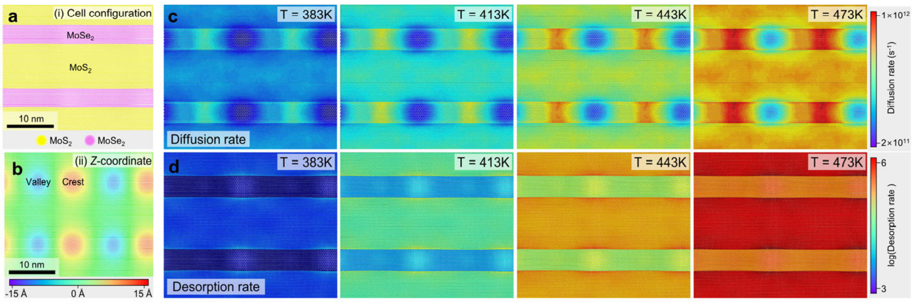

e

Temperature (K)		384	413	443	473
$k_{diffusion}$ (s^{-1})	max	6.26×10^{11}	7.77×10^{11}	9.23×10^{11}	1.07×10^{12}
	min	2.19×10^{11}	2.89×10^{11}	3.67×10^{11}	4.53×10^{11}
	max/min	2.86	2.69	2.52	2.37
$k_{desorption}$ (s^{-1})	max	7684.75	50402.2	252497	1017980
	min	955.151	6952.93	38165.3	166854
	max/min	8.05	7.25	6.62	6.10
$k_{adsorption}$ (s^{-1})		2793.33	2680.33	2587.99	2504.57

Supplementary Fig. 16. Diffusion and desorption rate varying with temperature. a, b Images of cell configuration (**a**) and *Z*-coordination (**b**) of MoS_2-$MoSe_2$ lateral superlattice in kMC simulation. **c, d** Visualizations of diffusion rate (**c**) and desorption rate (**d**) of TMA on the superlattice cell at T = 383, 413, 443, 473 K. **e** Table of rate constants of TMA in terms of temperature.

a

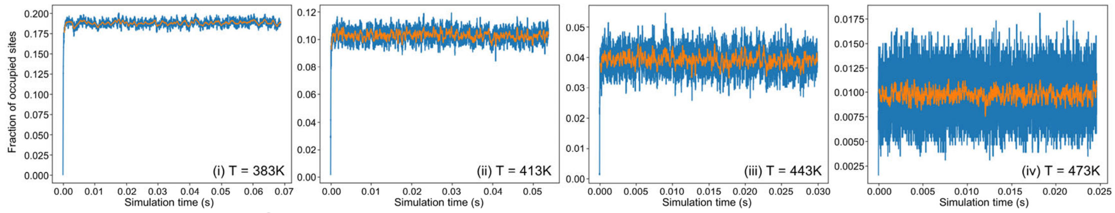

b

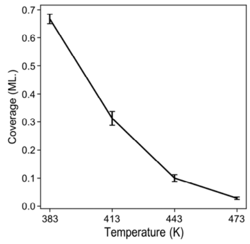

c

Temperature (K)	383	413	443	473
Average coverage (ML)	0.666	0.312	0.100	0.028
Simulation time in adsorption-desorption mode (ms)	125.186	41.090	26.690	24.142
Simulation time in diffusion mode (ns)	0.732	1.132	2.783	8.219

Supplementary Fig. 17. TMA equilibrium coverage obtained by kMC. a kMC simulations of TMA coverage varying with temperature (T = 383, 413, 443, and 474 K). The coverage is fraction of occupied chalcogen sites by TMA. **b** Average and standard deviations of simulation coverage of TMA obtained by adsorption-desorption mode in the kMC model. 1 ML has been defined as closed-packed TMAs occupying four chalcogen sites each. **c** Simulation time for average coverage of TMA in adsorption-desorption mode and diffusion mode.

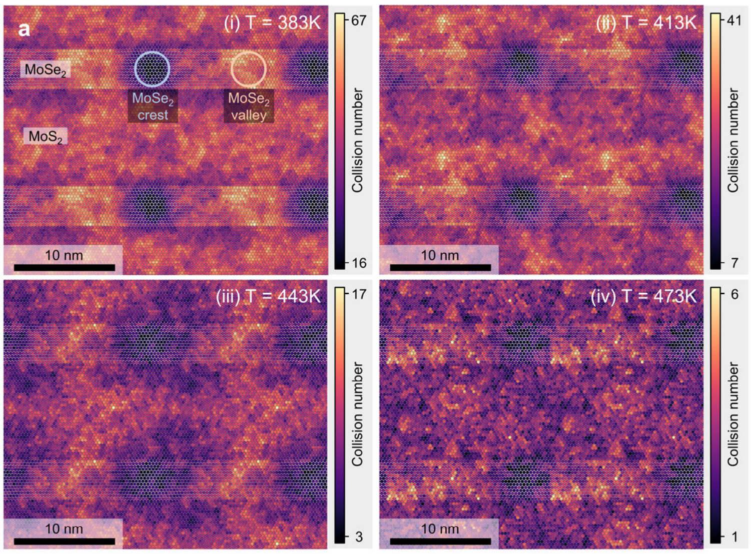

b

Temperature (K)	383	413	443	473
Total collisions in simulation	113641.83	63098.00	22293.27	7600.48
Minimum collisions	16.67	7.83	3.00	1.00
Maximum collisions	66.50	40.33	16.27	5.90
Average collisions	43.71	24.27	8.57	2.92
Average collision rate ($nm^{-2}s^{-1}$)	9.98×10^{11}	3.59×10^{11}	5.15×10^{10}	5.95×10^{9}

Supplementary Fig. 18. Heatmap of TMA collisions with varying temperature. a Visualization of collision number of TMA on the superlattice cell varying with temperature (T = 383, 413, 443, 474 K). **b** Table for total, min/max, average collisions, and average collision rate.

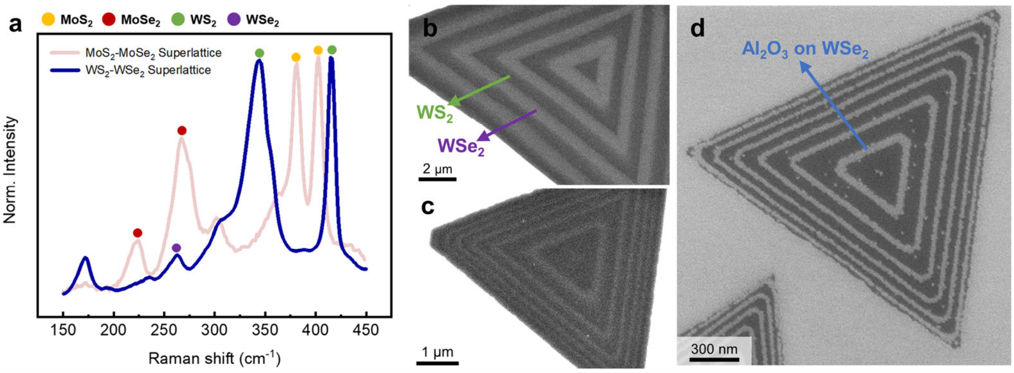

Supplementary Fig. 19. Al_2O_3 SAS-ALD on WS_2-WSe_2 lateral superlattice. a Raman spectrum of MoS_2-$MoSe_2$ (light red graph) and WS_2-WSe_2 (blue graph) lateral superlattices. The MoS_2, $MoSe_2$, WS_2, and WSe_2 peaks in the spectrum are indicated as yellow, red, green, and purple circles. **b, c** SEM images of WS_2-WSe_2 lateral superlattices grown by MOCVD process on Si/SiO_2 substrate. **d** SEM image of Al_2O_3 ALD (30 cycles) on the WS_2-WSe_2 superlattice.

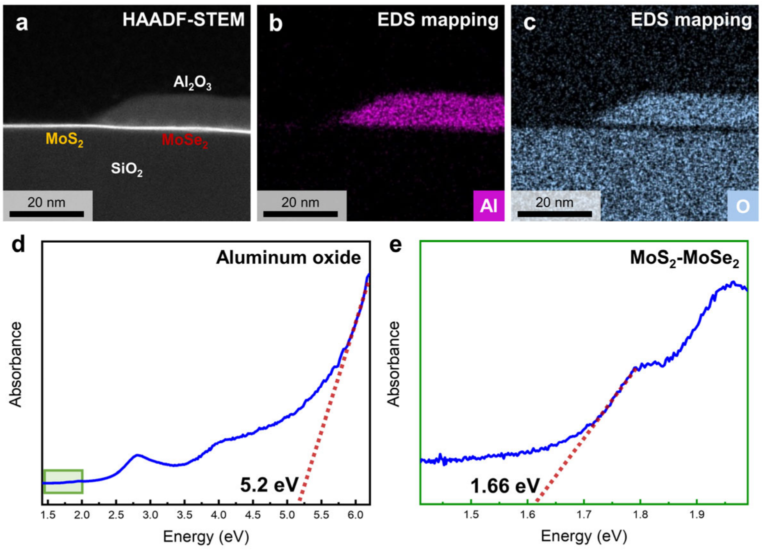

Supplementary Fig. 20. Interface quality and optical property of selectively deposited aluminum oxide. a-c Cross-sectional HAADF-STEM (**a**) and EDS mapping images (**b, c**) of aluminum oxide SAS-ALD structure on the MoS_2-$MoSe_2$ superlattice. EDS mapping images show aluminum (**b**, purple) and oxygen (**c**, blue) elements. **d** An absorption spectrum of aluminum oxide on the MoS_2-$MoSe_2$ lateral superlattice film by UV-VIS spectrophotometer. The bandgap of aluminum oxide is 5.2 eV in the Tauc plot. **e** The enlarged spectrum of green square region in **d**. The bandgap of MoS_2-$MoSe_2$ lateral superlattice film is 1.66 eV.

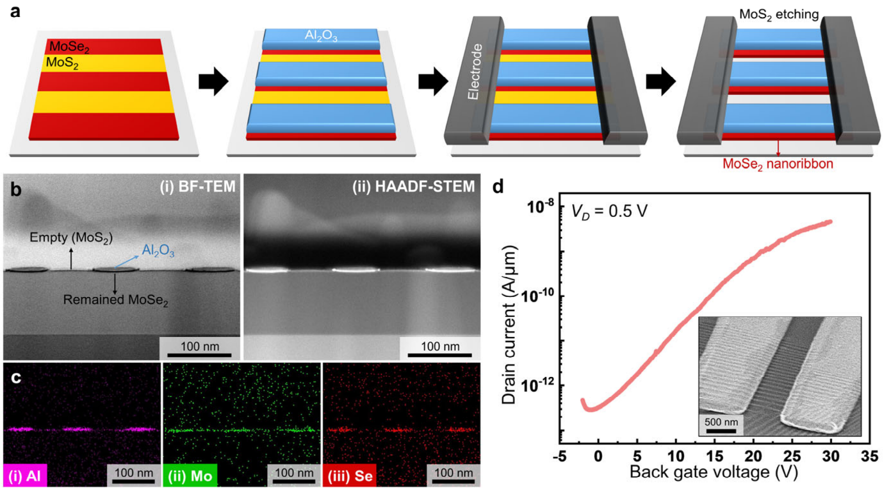

Supplementary Fig. 21. Electrical characterization of $MoSe_2$ nanoribbon FET (NRFET). **a** Schematic for fabrication of $MoSe_2$ NRFET device. **b, c** Cross-sectional BF-TEM, HAADF-STEM (**b**), and EDS mapping (**c**) images of $MoSe_2$ nanoribbon structure after RIE process onto Al_2O_3 SAS-ALD structure. **d** Transfer curve of $MoSe_2$ NRFET device. The inset shows the SEM image of the $MoSe_2$ NRFET device.

II. Supplementary Table

Process type	Reaction formula	$MoSe_2$ reaction site(s)		E_{rxn} (eV)	MoS_2 reaction site(s)		E_{rxn} (eV)
		site1	site2		site1	site2	
Adsorption	$TMA\ (g) + * \rightarrow TMA^*$	Se	/	-0.67 (-0.68)	S	/	-0.66
		V_{Se}	/	-0.48 (-0.50)	V_S	/	-0.48
	$H_2O\ (g) + * \rightarrow H_2O^*$	Se	/	-0.15	S	/	-0.15
		V_{Se}	/	-0.30	V_S	/	-0.25
Dissociative reaction	$TMA\ (g) + 2* \rightarrow DMA^* + CH_3^*$	Se	Se	2.03	S	S	1.59
		V_{Se}	Se	1.38	V_S	S	1.09
	$H_2O\ (g) + 2* \rightarrow OH^* + H^*$	Se	Se	5.22	S	S	5.09
		V_{Se}	Se	1.29	V_S	S	1.22

Supplementary Table 1. Adsorption and reaction energies on TMD surface sites. Energies (E_{rxn}) for potential adsorption reactions on transition dichalcogenide (TMD) surface. In the reaction formula, asterisk (*) indicates the adsorption site. The type of adsorption site is denoted in the reaction site(s) column, where Se, V_{Se}, S, and V_S represent the Se top, Se vacancy, S top, and S vacancy site in $MoSe_2$ and MoS_2 surfaces, respectively. For dissociative reactions, the sites are numbered according to their appearance in the right-hand side of the reaction formula. Adsorption on -3 % lattice strained $MoSe_2$ surface is indicated in parentheses. The precursors are trimethyl aluminum (TMA), dimethyl aluminum (DMA), and H_2O.

III. Supplementary Notes

Supplementary Note 1. Descriptions of CAS-ALD researches

Area-selective ALD (AS-ALD) is a method of selectively depositing a target material only on the desired region by creating a barrier which can give a difference in deposition rate[1-4]. For example, CH_3 or CF_3 tail group of a self-assembled monolayer (SAM)[1-3,5-9], surface termination such as Si-H[10-14], inherent selectivity in metal-insulator combination[4,15-18], and non-dangling bond basal plane of 2D materials[19-23] can be used as a conventional AS-ALD (CAS-ALD) barrier. These barriers suppress chemisorption or reaction of specific precursors and prevent material deposition in a specific region. That is, by forming a barrier which is inert to a chemical reaction with the ALD precursors, deposition can only occur in the region without the barrier.

However, even if chemisorption of a specific precursor is prevented, material deposition can still occur on the barrier through physisorption due to van der Waals force between surface and precursors or penetration of precursors into barrier materials[2,3,5,20,24]. Many researches attempted to reduce the undesired deposition by physisorption through modification of process pressure or precursor types[2,3,5,13,14,24,25], but fundamental elimination of such defects is challenging. In particular, it is well known that highly reactive and small-sized (low steric hinderance) precursors, such as trimethyl aluminum (TMA), can easily penetrate the barrier[5,13,24], resulting in many defects. Furthermore, CAS-ALD needs pre-patterning process of substrate or barrier material by top-down lithography, which means resolution of CAS-ALD is limited by optical source[26,27]. Additionally, when the barrier materials, such as SAM molecule, are formed in a small area of a few nm scale, they cannot maintain their structures, leading to the pattern collapse and resolution degradation[28-30].

Supplementary Note 2. Characterization of MoS_2, $MoSe_2$ regions in lateral superlattice

Supplementary Fig. 1d is HAADF-STEM image of the MoS_2 and $MoSe_2$ regions in the lateral superlattice. In the interface region between MoS_2 and $MoSe_2$ (Supplementary Fig. 1d (i) and (ii)), we can observe 10 nm $MoSe_2$ region (light gray), and a sharp $MoSe_2$-$MoSe_2$ interface. Within the MoS_2 area (Supplementary Fig. 1d (iii)), molybdenum vacancy (V_{Mo}) is not detected. However, in the sulfur site, we observe Mo_S, Se_S, S_S, and V_S, with a progressively darker appearance. Similarly, within the $MoSe_2$ area (Supplementary Fig. 1d (iv)), V_{Mo} doesn't exist, but in the selenium site, we find Se_{Se}, S_{Se}, and V_{Se}. These sites are indirectly inferred defects based on the relative brightness difference from the Mo_{Mo} site. While it is not easy to precisely identify the specific types of individual sites, it is clear that various types of defects (V_S, V_{Se}, S_{Se}, Se_S, Mo_S) are expected at the chalcogen sites in CVD-grown lateral superlattice. Importantly, these defects at various chalcogen sites are randomly distributed within each MoS_2 and $MoSe_2$ areas and do not exhibit any periodic arrangement. This suggests that the defects do not play a significant role in SAS-ALD, and indeed, when analyzing TMA's reaction with the chalcogen vacancy (major defect in our superlattice), SAS-ALD can't be explained by reaction or defect sites.

Supplementary Note 3. Periodic deposition of Al_2O_3 on $MoSe_2$ ripple and buckle

In the MoS_2-$MoSe_2$ lateral superlattice, the height of the out-of-plane deformation in the $MoSe_2$ region (primarily) varies with the width of $MoSe_2$. Supplementary Fig. 1e shows the AFM images of the superlattice with small $MoSe_2$ width (less than 100 nm). In these images, the $MoSe_2$ ripple heights are 0.2, 0.3, and 0.5 nm, where the $MoSe_2$ widths are 20, 50, and 60nm, respectively. However, as shown in Supplementary Fig .4a, when the $MoSe_2$ width becomes wider (more than 100 nm), it exhibits high buckle height of 3 nm. These ripple or buckle occur due to the lattice mismatch between MoS_2 and $MoSe_2$ and periodically exist within the $MoSe_2$

regions.

When performing our SAS-ALD on these lateral superlattices, Al_2O_3 islands are periodically deposited similar to the periodicity of ripple or buckle. As seen in Figs. 3a and 3h, when the $MoSe_2$ width is small, Al_2O_3 islands are positioned in alignment with the ripple periodicity. Furthermore, as observed in Supplementary Figs. 4b and 4d, when the $MoSe_2$ width is large, periodic deposition around buckles and at the interfaces can be confirmed. Importantly, when both wide and narrow $MoSe_2$ widths coexist, as shown in Supplementary Fig. 4e, each deposition trend is observed together. Theses periodic deposition clearly demonstrate that SAS-ALD is not solely a result of surface difference between MoS_2 and $MoSe_2$ but is significantly associated with areas where deposition occurs preferentially within $MoSe_2$.

When the $MoSe_2$ width is narrow (< 50 nm), Al_2O_3 is primarily deposited in the valley region of the $MoSe_2$ ripple structure. In contrast, for a larger $MoSe_2$ width (> 200 nm), initial nucleation occurs not only around the $MoSe_2$ buckle region but also in the interface regions between MoS_2 and $MoSe_2$. These two initial nucleation sites will be related to our SAS-ALD mechanism, which exhibits selectivity in adsorption and surface diffusion of the ALD precursors. In the case of a narrow $MoSe_2$ width, the distance between the $MoSe_2$ valley region and the interface region is very close (< 50 nm), allowing the ALD precursors to predominantly move to the most stable region, the $MoSe_2$ valley site. However, as the $MoSe_2$ width increases, the distance between the $MoSe_2$ buckle and the interface region widens (> several hundred nm). This can cause the ALD precursors, which are moving around the interface region and not sufficiently reaching the $MoSe_2$ buckle region, to nucleate not necessarily at the most preferred buckle but rather in a reasonably preferred interface region. In other words, depending on the width of the superlattice, initial nucleation can occur not only on the compressed $MoSe_2$ region, the energetically most stable sites, but also on the MoS_2-$MoSe_2$ interface regions.

Supplementary Note 4. MD simulation strain mapping in triangular cell

Fig. 3d and Supplementary Fig. 5a represent strain mapping simulated in rectangular superlattice cells, showing strain in *x*-direction, *y*-direction, and the *x* and *y* direction average, respectively. The strain distribution in each cell can be observed in Supplementary Figs. 5b-d, and it is evident that, unlike MoS_2 where strain is almost released, $MoSe_2$ retains significant compressive and tensile strains in the *x*-direction.

It is also of importance to analyze the effect of transition metal chalcogenides (TMDs) under its characteristic triangular growth. We model three different situations where $MoSe_2$:MoS_2 is 1:2.5 (~ 6:15 nm), 1:2 (~ 6.75:13.5 nm), and 1:1 (9:9 nm) in triangular repetitive structures (Supplementary Figs. 5e-k). In the *Z*-height images, larger $MoSe_2$ width results in higher ripple heights (Supplementary Fig. 5i). In terms of average strain, as the width of the $MoSe_2$ becomes smaller, the abundance of Se atoms with higher compressive/tensile strain increases due to the formation of ripples on top of a larger lattice mismatch (Supplementary Fig. 5f). The effect can also be seen in Supplementary Fig. 5k, where the density of $MoSe_2$ near 0 % strain decreases and the density in both extrema increases as the width of $MoSe_2$ decreases (shown in red lines). On the contrary, with a larger $MoSe_2$ region, more S atoms with a bigger tensile strain appear, as can be seen from the shifting of the MoS_2 peak towards the right (shown in yellow lines). Comparing the two simulations (rectangular superlattice vs triangular superlattice), the distribution of average strain in the $MoSe_2$ region of the triangular superlattice shows a much clearer bimodal distribution, which can be attributed to the smaller width of each region and the bigger confining effects of the triangular MoS_2 acting simultaneously on both *x* and *y* directions.

Supplementary Note 5. Reaction and defect effects in SAS-ALD

In CAS-ALD, the difference in reaction rate (or chemisorption) is the main cause of selective deposition. Therefore, we also need to analyze the impacts of reaction and defects in our SAS-ALD mechanism.

(i) Reaction between ALD precursor and TMD non-defect surface

Supplementary Fig. 6a schematically illustrates the dissociative reaction between TMA and the MoS_2 ($MoSe_2$) surface. TMA can react at various sites on the MoS_2 ($MoSe_2$) surface, including chalcogen, chalcogen bridge, and hollow sites. The most energetically stable reaction of TMA occurs at the chalcogen bridge in our simulation, where TMA reacts by removing one methyl group and bonding to form dimethyl aluminum (DMA). In this reaction, the reaction energy for TMA-MoS_2 is 1.59 eV, and for TMA-$MoSe_2$, it is 2.03 eV, both of which are non-spontaneous endothermic reactions (Supplementary Table 1). Considering that the energy involved in typical ALD reaction is very low (e.g., -0.9 eV in the TMA-H_2O reaction in Supplementary Fig. 11f), the reaction between TMA and TMD surface can be considered an extremely slow reaction. Furthermore, the lower reaction energy for TMA-MoS_2 compared to TMA-$MoSe_2$ means that the reaction and Al_2O_3 deposition are more likely to occur on the MoS_2 region. This contrasts with the results of our SAS-ALD where the deposition region is $MoSe_2$.

We have also investigated the chemisorption of TMA on the basal plane. Supplementary Fig. 7 depicts the charge density difference plot of TMA adsorbed on $MoSe_2$ (Supplementary Figs. 7a-c) and on MoS_2 (Supplementary Figs. 7d-e) with different lattice strain induced. It is found that the electron transfer between the TMA and TMD surface is minimal, with the plotted iso-value for the charge density difference being only $0.001 e/bohr^3$. Also, strain-induced change between the different strained cases is quite minimal, with marginally larger iso-surfaces towards tensile strained cases. Supplementary Figs. 7a and 7d are TMD under compressive

strain, and Supplementary Figs. 7c and 7f are TMD under tensile strain. Considering that the adsorption energy is stronger under compressive strain than tensile strain, the smaller iso-surface in Supplementary Figs. 7a and 7d indicates that the source of stronger adsorption in the compressed TMD region is not through chemical bonding character between the aluminum and the chalcogen atoms, but rather the van der Waals interaction between the precursor and the TMD.

(ii) Reaction between ALD precursor and TMD chalcogen vacancy sites

As shown in HAADF-STEM image in Supplementary Fig. 1d, various types of defects are present, but do not present periodicity. As discussed in the main script, the initial nucleation presents such periodicity (See Fig. 3a and Supplementary Fig. 4e). Thus, it is quite evident that the defects are not the cause of SAS-ALD. Nonetheless, among the various defects, chalcogen vacancy sites (V_S, V_{Se}) are predominant in our superlattice, and we have also analyzed the reaction between these vacancy sites and ALD precursors. Supplementary Fig. 6b schematically illustrates the reaction where TMA removes one methyl group and bonds with DMA on a chalcogen vacancy site. In this reaction, both TMA-V_S and TMA-V_{Se} exhibit high reaction energy, 1.09 eV and 1.38 eV, respectively, indicating a relatively slow reaction (Supplementary Table 1). Furthermore, since the reaction energy is lower at V_S than V_{Se}, if the governing factor of nucleation is through these vacancies, the Al_2O_3 deposition should occur more easily on the MoS_2 region, contrary to our case. Moreover, the reaction of H_2O with the chalcogen sites, also show a reversed trend, with H_2O-V_S (1.21 eV) and H_2O-V_{Se} (1.28 eV), suggest that the reactions are more likely to occur on the MoS_2 region. Hence, even in the case of chalcogen vacancy sites, the results contradict those observed in our SAS-ALD experiments. The adsorption energy of TMA on chalcogen vacancy sites has also been calculated. On the MoS_2 surface, TMA's adsorption energy is -0.66 eV, but on V_S, it is -0.48 eV, indicating even less stable adsorption on vacancy site than non-defected MoS_2 surface. Also, on the $MoSe_2$

surface, adsorption energy is -0.67 eV, but on V_{Se}, it is -0.48 eV. Therefore, TMA's adsorption is not stabilized on the chalcogen vacancy sites, again suggesting that vacancies don't have impact on the SAS-ALD mechanism.

(iii) Undesired deposition on multiple vacancies

Considering the two situations mentioned above, it is clear that neither reactions nor chalcogen vacancies can explain our SAS-ALD. However, it has been confirmed that specific defects can lead to undesired deposition in SAS-ALD. Supplementary Fig. 8a shows SEM images for Al_2O_3 AS-ALD on a MoS_2-$MoSe_2$ lateral superlattice (i) immediately after CVD growth, (ii) 7 days later, and (iii) 14 days later. The superlattice was stored under ambient air condition. As seen in the SEM images, it is evident that the longer the superlattice is exposed in the air, the more undesired Al_2O_3 deposition occurs on the MoS_2 region.

The cause of this selectivity loss was analyzed through TEM measurements. Supplementary Figs. 8b-d are HAADF-STEM images of the superlattice that was not degraded in air condition. In the interface area (Supplementary Fig. 8c) and in each MoS_2 and $MoSe_2$ region (Supplementary Fig. 8d), similar to Supplementary Fig. 1d, there are slight chalcogen vacancies, but no significant defects are found. However, in the superlattice that degraded over 14 days under air conditions, many large multiple vacancies were observed at the interface and in each MoS_2 and $MoSe_2$ region (red circles in Supplementary Figs. 8e-g). These large multiple vacancies, resulting from the degradation of lateral superlattice, occur in both MoS_2 and $MoSe_2$ areas, are found to cause the undesired deposition in SAS-ALD. Importantly, as the defect size is a few nanometer scales, the SiO_2 substrate underneath is exposed, making it highly likely for TMA to reaction in these regions. However, it is evident that these large defects, present only in the degraded superlattice, are significantly different from our SAS-ALD process described in the main script.

Supplementary Note 6. The adsorption energy trend for various precursors and temperature

In addition to TMA, the adsorption energies of other various precursors need to be considered.

(i) H_2O

Supplementary Fig. 11 explains the adsorption and reaction of H_2O with TMA. As evident in Supplementary Table 1 and Supplementary Figs. 11a and 11b, H_2O, unlike TMA, exhibits highly unstable adsorption energies on MoS_2 and $MoSe_2$ surfaces (-0.15 eV on MoS_2, -0.15 eV on $MoSe_2$). H_2O molecules have a slightly more stable energy on the $MoSe_2$ side but are still highly unstable for adsorption on TMD surface, meaning almost all H_2Os are removed during H_2O purge time. Even at chalcogen vacancy sites (V_S, V_{Se}), where H_2O shows slightly more stable adsorption energies (-0.25 eV on V_S, -0.30 eV on V_{Se}), H_2O molecule is highly unstable for adsorption compared to TMA. Interestingly, adsorption of H_2O on pre-adsorbed TMA (Supplementary Fig. 11b) is the most stable among other sites (-0.43 eV in Supplementary Fig. 11c) where the TMA acts as a Lewis acid site. Furthermore, the adsorption energy of H_2O on pre-adsorbed TMA becomes even more stabilized with compressive strain on the TMD (-0.44 eV in Supplementary Fig. 11c). In this TMA-H_2O complex, we performed a CI-NEB calculation for the reaction in which a methyl group is removed and forms dimethyl aluminum hydroxide (DMAOH). It is found that this reaction has a low activation energy (0.34 eV in MoS_2, 0.35 eV in $MoSe_2$) and is highly endothermic (-0.944 eV in MoS_2, -0.933 eV in $MoSe_2$), indicating that the reaction between pre-adsorbed TMA and H_2O is very fast. This is comparable to the previously reported first TMA reaction on hydroxylated Al_2O_3 surface where the activation energy was 0.30 eV.[31] In summary, H_2O adsorption itself has minimal impact on SAS-ALD, but the rapid reaction of H_2O with pre-adsorbed TMA suggests that site selectivity of TMA is a key factor in SAS-ALD.

(ii) Various ligands for precursor of Al_2O_3, Sb_2Se_3, HfO_2, and Te

Unlike TMA and H_2O used in Al_2O_3 ALD, it is not easy to perform accurate simulations for other ALD processes that use very bulky precursors. However, in SAS-ALD, all precursors are expected to adsorb primarily through van der Waals interactions. In such cases, the bulky ligands will have a significant influence on adsorption compared to the central metal. Therefore, in Supplementary Fig. 12, the interactions of the hydrogenated ligands of each precursor on the TMD surface are compared instead of the bulky precursors themselves.

Supplementary Fig. 12a (i) shows the adsorption energy of CH_4, hydrogenated form of CH_3 which is the ligand part of TMA. There is some difference compared to the adsorption energy trend of TMA in Fig. 4b, but the conclusion that adsorption on the compressed $MoSe_2$ region (pink bar) is the most stable among the three cases remains the same. The difference is thought to be due to the influence of factors such as symmetry between the lattice and the precursor that is not present in ligand-only calculation. In addition, Supplementary Figs. 12a (ii)-(iv) show the adsorption energy of the hydrogenated ligands of Sb_2Se_3, HfO_2, and Te precursors, respectively, and in all cases, adsorption on compressed $MoSe_2$ (pink bar) is the most stable. Through confirmation of the adsorption energy of these ligands, it can be expected that all the various precursors in SAS-ALD prefer the compressed $MoSe_2$ region to be adsorbed.

(iii) Precursor varying with metals and ligands

We examined other possible precursors with different metals and ligands to find trends in their behavior in Supplementary Fig. 13. We calculated the adsorption energy on lattices under 0 % and -3 % strain on $MoSe_2$ and MoS_2. Across the three group 13 metals (denoted TMA, TMGa, TMIn for Al, Ga, In), all results show lattice compression leads to stronger adsorption energy, with adsorption on $MoSe_2$ being slightly stronger. Across the different ligands (methyl, ethyl, and propyl ligands, denoted TMA, TEA, TPA), longer chains show stronger adsorption behavior, as expected due to the dominant role of van der Waals forces in adsorption. Moreover,

even with different ligands, the adsorption energy of the precursors (TMA, TEA, TPA) becomes more stabilized when the TMD is under compressive strain. This indicates that many precursors are most stable when adsorbed on the compressed $MoSe_2$ valley region.

(iv) Gibbs free energy for adsorption of TMA

The above adsorption energies are all enthalpy-based energies, making it difficult to assess their temperature dependence. Supplementary Fig. 14 shows the Gibbs free energy plot for TMA adsorption calculated at T = 300 K and T = 500 K. The absolute value of adsorption energy increases with higher temperatures, making adsorption less stable. However, even when the temperature factor is considered, TMA adsorption is further stabilized under compressive strain in TMD surface, which is also apparent in the desorption rate visualization in Supplementary Fig. 16d.

Supplementary Note 7. Additional information on kinetic Monte Carlo simulation

(i) Determination of the reaction rates on MD simulated superlattice

Though the DFT calculations give us a guess of the mechanism of the SAS-ALD, adsorption energies are vague in that the exact kinetics of ALD has not been uncovered. The lattice kMC simulation of TMAs on the TMD surface was carried out with the aim of proving the validity of our mechanism, providing numerical evidence within a real-world context.

In setting up of the kMC simulation, we made several assumptions to estimate rates for each site on a superlattice surface obtained by the MD simulations. Given the unique atomic strain experienced by each atom in MD simulations, it became essential to derive rates for each distinct atomic environment. While methods such as cluster expansion or machine learning potentials are capable of this, they require extensive computational efforts. To maintain simplicity, we employed basic assumptions in estimating adsorption, desorption, and diffusion rates, which proved sufficient for demonstrating the structure-energy-rate dependencies in our

specific scenario. Our focus was exclusively on the initial trimethylaluminum (TMA) pulse on a clean transition metal dichalcogenide (TMD) surface.

The assumptions underlying our model are as follows:

1. In obtaining the vibrational contributions to the ZPE and entropy, we treat imaginary and real frequencies smaller than 50 cm^{-1} to 50 cm^{-1}, as small vibrational frequencies can lead to large errors in entropy.
2. We determined Gibbs free energy for TMA adsorption and diffusion using the Hindered-Rotor-Translator (HRT) approximation for surface-adsorbed species and the ideal gas approximation for the gas phase. In the HRT approximation, we applied constant values for rotational barriers.
3. We limited our scope to three crucial reactions: TMA adsorption, desorption, and first-neighbor hopping (diffusion).
4. Adopting the principles of kinetic gas theory, we assumed that a uniform collision with the surface sites leads to barrier-less adsorption.
5. For simplicity, curvature effects on adsorption, desorption, and diffusion are neglected, and presumed to be related by the strain tensors and nearest atom distances.
6. Prefactor for diffusion rate is fixed to 1×10^{13} (s^{-1}).

Using these assumptions, we calculate 8 different structures' adsorption Gibbs free energies and 8 different diffusion barriers on tensile and compressive strained $MoSe_2$ and MoS_2 surfaces (4 for each). We choose linear regression to relate the MD obtained conformational information and DFT obtained thermodynamics. The resulting linear fitting is depicted in Supplementary Figs. 14a-c, which shows a reasonable match in adsorption Gibbs free energies the $(\epsilon_{xx} + \epsilon_{yy} + \epsilon_{zz})/3$ -0.04 ~ 0.02 range (corresponding to planar lattice compression of -10 % ~ +3 %), and chalcogen spacing with diffusion barriers. Also, the adsorption and desorption rates are comparable to reference from which we follow the methods for calculation of rates, where the adsorption rate is 5.2×10^3 and 4.2×10^3, desorption rate is 7.4×10^2 and 5.6×10^9 (s^{-1}) in 298

and 450 K, respectively[31]. In order to transfer the fitted model to our simulation, we carried out an MD simulation with a small cell of 17 nm width and 14 nm height, with $MoSe_2/MoS_2$ ratio of 4/10. From the obtained geometry we compute for each top-site chalcogen atom their local strain (for estimating desorption rates) and an ID representing the average distance from its neighboring chalcogen atoms. The ID is obtained by digitizing the distance to 40 groups (20 for Se and S each). We then use the ID pairs of neighboring atoms to obtain the diffusion rates from a precomputed table.

From the visualized rates it is interesting to note that with higher temperatures, the diffusion and desorption rates increase, where the latter increases by 5 times every 30 K (see Supplementary Figs. 16a-e). This results in vastly different surface coverage in our simulations for each temperature. It should also be noted that while the max/min ratio of the rates decreases with increasing temperature, the values are still above 2 and 6 for diffusion and adsorption, indicating vastly different phenomena can happens at the $MoSe_2$ valley (fast diffusion, slow desorption), $MoSe_2$ crest (slow diffusion, moderate desorption), and MoS_2 (moderate diffusion, very fast desorption) regions.

(ii) Obtaining the heatmap of properties from the kMC simulation

In running the kMC simulation, we face a problem as the reaction rates between adsorption/desorption and diffusion differs by up to 6 orders of magnitude. A kMC considering all three reactions at the same time will lead to almost all selected events being diffusion. There exist methods to sample the rare events such as rate scaling, where high rate events are suppressed after every equilibration[32-34]. But such methods are used for lattice kMC simulations with fixed geometries, which is different from our case where there exist much too many states with all adsorption sites being different from one another. To circumvent this situation, we conducted the experiments in two different modes as explained in the MD simulation, DFT calculation, and kMC simulation section of Methods in the main script, where we run

adsorption/desorption-only simulation (ads-des mode) followed by diffusion-only simulation (diff mode). In the former, we take the last 50 % of the simulation frames to obtain the ads-des mode occupation heatmap. In the latter, we take the last 70 % of the simulation frames to obtain the diff mode occupation heatmap, and also measure the collision frequencies occurring on each chalcogen site, where the collision has been defined as an event where a TMA diffuses towards the first neighbor site of another TMA. This estimation is based on the expectation that the reactive $Al(OH)_n(CH_3)_{3-n}$ intermediates, formed after the initial H_2O pulse, will exhibit diffusion rates comparable to those of the structurally similar TMAs. The dimeric aluminum intermediates, resulting from the collision of these intermediates will possess significantly decreased diffusion and desorption rates compared to the single Al intermediates, primarily due to enhanced van der Waals (vdW) interactions as a result of the increased mass of the intermediate. Consequently, the collision probability and dynamics involving monomeric aluminum intermediates at various lattice sites may provide insights into the likely positions of subsequent nucleation processes. Within the two simulations, we obtained the equilibrium coverage, occupation and collision numbers of different sites, depicted in Fig. 4c and Supplementary Figs. 17-18. We initially run 6 independent simulations at each different temperature to obtain an average of the properties, and sample 5 and 15 more runs for 443 K and 473 K, until all surface sites have experienced at least one collision. From Supplementary Fig. 17a, we find that the simulation reaches equilibrium coverage within 10 % of the simulation steps. Moreover, the equilibrium coverage decreases exponentially from 0.667 to 0.028 ML in 383 K to 473 K, respectively. As a result, the number of collisions in the diffusion mode also decreases significantly, with the collision rate decreasing more than an order of magnitude. When considering the desorption rate along with this fact, such lowering of the collision rate demonstrates how selectivity is lifted in higher temperatures, as the particles have less probability of forming nucleates before reaching the pre-formed nucleates (possibly from

the ALD cycle before), most probably located at the $MoSe_2$ basin.

Our simulation demonstrates that the morphology of the substrate will affect the diffusion rates, thereby changing the probable location of the ALD nucleation. However, it should be noted that this is a model system, different from the real-world scenario in many aspects. For example, our model uses a fixed substrate morphology, while thermal effects will affect not only the adsorption/desorption/diffusion rates, but also the substrate morphology itself in real cases. Moreover, due to computational limits, our simulation cell has been set to ~204 nm^2 in area, which is much smaller than the experimental situation or the MD simulation in previous sections. Thus, size effects are present. Indeed, such can be seen from the collision map of Fig. 4c, where some regions in MoS_2 also have high collision rates. This is presumably due to the fact that there exists a curvature induced on the MoS_2 region which is propagated from the ripples in $MoSe_2$ region. Indeed, such effects will only be present near the edges of the $MoSe_2$ regions if a larger simulation cell is employed, or if substrate to the superlattice is introduced, where the substrate-superlattice interaction will flatten the region to an extent.

Nevertheless, the kMC simulations provide valuable insights into the SAS-ALD where we find the extent of temperature-coverage dependence, as well as occupation and collision probability on different regions of the superlattice.

Supplementary Note 8. Interface and optical property of selectively deposited aluminum oxide

We discussed the selective deposition of various materials on the 2D lateral superlattices. To increase a scalability of our method, it is necessary to analyze the characteristics of the selectively deposited materials.

First, we analyzed the interface quality between the deposited materials and the 2D surface. Supplementary Fig. 20a is a cross-sectional HAADF-STEM image showing the selectively

deposited aluminum oxide on the $MoSe_2$ region. The white line in the image corresponds to the MoS_2-$MoSe_2$ surface, and the bright gray region above the white line represents aluminum oxide. We observed that there are no serious defects such as cavities at the interface between $MoSe_2$ and aluminum oxide, indicating a flat interface with good contact quality. Moreover, the clear image of the $MoSe_2$ suggests that AS-ALD does not cause degradation to the 2D surfaces. Supplementary Fig. 20b and 20c show EDS mapping images of the SAS-ALD structure, representing the distribution of aluminum and oxygen elements, respectively. The selectively deposited aluminum oxide region exhibits a same element signal in both areas, near and far from the interface. Thus, the quality of aluminum oxide at the interface is expected to be identical to that in the bulk. The images observed through STEM indicate a clean and uniform interface between the 2D material and selectively deposited oxide.

Next, we measured the optical properties of the selectively deposited aluminum oxide on the $MoSe_2$ region. Supplementary Fig. 20d shows an absorbance Tauc plot measured by UV-VIS spectrophotometer. A monolayer MoS_2-$MoSe_2$ lateral superlattice film was grown on the Si/SiO_2 substrate and transferred onto a transparent fused silica substrate. Lastly, aluminum oxide was deposited on the superlattice film by ALD (100 cycles). Typically, an amorphous aluminum oxide has a bandgap of 5 to 7 eV range. In our case, the bandgap of the aluminum oxide, extracted from the Tauc plot in Supplementary Fig. 20d, is 5.2 eV, which is in valid range of bandgap. Supplementary Fig. 20e is an enlarged absorbance spectrum of the green square region in Supplementary Fig. 20d, revealing that the bandgap of the MoS_2-$MoSe_2$ film is 1.66 eV. Through the aforementioned analyses, we can confirm that the material deposited by the SAS-ALD technique exhibits a good interface on the 2D surface and demonstrates valid properties.

Supplementary Note 9. Fabrication of $MoSe_2$ nanoribbon FET (NRFET)

We have fabricated a $MoSe_2$ nanoribbon field-effect transistor (NRFET), using Al_2O_3 on the $MoSe_2$ as a hard mask for dry etching, and have subsequently measured its electrical properties, to demonstrate the potential capability of fabricating novel device structures using our SAS-ALD method. Supplementary Fig. 21a schematically shows the fabrication process of the $MoSe_2$ NRFETs. After growing the MoS_2-$MoSe_2$ lateral superlattice, Al_2O_3 AS-ALD (60 cycles) was performed. Subsequently, source/drain electrodes were deposited by using electron-beam lithography to make contact with the MoS_2 surface. Finally, the exposed MoS_2 area in the channel region was removed through reactive ion etching (RIE) process to fabricate $MoSe_2$ NRFETs. Supplementary Fig. 21b presents TEM results after removing MoS_2 region by RIE, showing the $MoSe_2$ nanoribbon. As confirmed by bright-field TEM (BF-TEM) and high angle annular dark field STEM (HAADF-STEM), $MoSe_2$ beneath the Al_2O_3 remains intact, while the MoS_2 regions where Al_2O_3 was not deposited have been removed. This is also evident in the cross-sectional EDS mapping images in Supplementary Fig. 21c, showing the molybdenum element is removed in the previous MoS_2 region.

We conducted basic electrical characterization of the $MoSe_2$ NRFET, which is shown in the inset image of Supplementary Fig. 21d. Supplementary Fig. 21d shows a transfer curve of nanoribbon $MoSe_2$ channels, which was measured with a drain voltage of 0.5 V. The on/off ratio is about 2×10^4, the mobility is 0.04 $cm^2/V\cdot s$, and the subthreshold swing (SS) is 5.5 V/dec.

The FET performance may not be outstanding, but it is evident that gating properties are sufficiently confirmed even in narrow nanoribbons with widths in the tens of nanometers.

IV. Supplementary References

1 Bobb-Semple, D., Nardi, K. L., Draeger, N., Hausmann, D. M. & Bent, S. F. Area-selective atomic layer deposition assisted by self-assembled monolayers: a comparison of Cu, Co, W, and Ru. *Chem. Mater.* **31**, 1635-1645 (2019).

2 Parsons, G. N. & Clark, R. D. Area-selective deposition: fundamentals, applications, and future outlook. *Chem. Mater.* **32**, 4920-4953 (2020).

3 Mackus, A. J. M., Merkx, M. J. M. & Kessels, W. M. M. From the bottom-up: toward area-selective atomic layer deposition with high selectivity. *Chem. Mater.* **31**, 2-12 (2019).

4 Cao, K., Cai, J. & Chen, R. Inherently selective atomic layer deposition and applications. *Chem. Mater.* **32**, 2195-2207 (2020).

5 Seo, S. *et al.* Reaction mechanism of area-selective atomic layer deposition for Al_2O_3 nanopatterns. *ACS Appl. Mater. Interfaces* **9**, 41607-41617 (2017).

6 Hashemi, F. S. M. & Bent, S. F. Sequential regeneration of self-assembled monolayers for highly selective atomic layer deposition. *Adv. Mater. Interfaces* **3**, 1600464 (2016).

7 Hashemi, F. S. M., Prasittichai, C. & Bent, S. F. A new resist for area selective atomic and molecular layer deposition on metal–dielectric patterns. *J. Phys. Chem. C* **118**, 10957-10962 (2014).

8 Sampson, M. D., Emery, J. D., Pellin, M. J. & Martinson, A. B. F. Inhibiting metal oxide atomic layer deposition: beyond zinc oxide. *ACS Appl. Mater. Interfaces* **9**, 33429-33436 (2017).

9 Lee, B. H. *et al.* Low-temperature atomic layer deposition of copper metal thin films: self-limiting surface reaction of copper dimethylamino-2-propoxide with diethylzinc. *Angew. Chem. Int. Ed.* **48**, 4536-4539 (2009).

10 McDonnell, S. *et al.* Controlling the atomic layer deposition of titanium dioxide on silicon: dependence on surface termination. *J. Phys. Chem. C* **117**, 20250-20259 (2013).

11 Methaapanon, R. & Bent, S. F. Comparative study of titanium dioxide atomic layer deposition on silicon dioxide and hydrogen-terminated silicon. *J. Phys. Chem. C* **114**, 10498-10504 (2010).

12 Hackley, J. C., Gougousi, T. & Demaree, J. D. Nucleation of HfO_2 atomic layer deposition films on chemical oxide and H-terminated Si. *J. Appl. Phys.* **102** (2007).

13 Kim, H. G. *et al.* Effects of Al precursors on deposition selectivity of atomic layer deposition of Al_2O_3 using ethanethiol inhibitor. *Chem. Mater.* **32**, 8921-8929 (2020).

14 Xu, W. *et al.* Functionalization of the SiO_2 surface with aminosilanes to enable area-selective atomic layer deposition of Al_2O_3. *Langmuir* **38**, 652-660 (2022).

15 Singh, J. A. *et al.* Area-selective atomic layer deposition of metal oxides on noble metals through catalytic oxygen activation. *Chem. Mater.* **30**, 663-670 (2018).

16 Tao, Q., Jursich, G. & Takoudis, C. Selective atomic layer deposition of HfO_2 on copper patterned silicon substrates. *Appl. Phys. Lett.* **96**, 192105 (2010).

17 Kannan Selvaraj, S., Parulekar, J. & Takoudis, C. G. Selective atomic layer deposition of zirconia on copper patterned silicon substrates using ethanol as oxygen source as well as copper reductant. *J. Vac. Sci. Technol. A* **32**, 010601 (2014).

18 Junige, M. *et al.* Area-selective atomic layer deposition of Ru on electron-beam-written Pt(C) patterns versus SiO_2 substratum. *Nanotechnology* **28**, 395301 (2017).

19 Kim, K. *et al.* Selective metal deposition at graphene line defects by atomic layer deposition. *Nat. Commun.* **5**, 4781 (2014).

20 Zhang, H. *et al.* Nucleation and growth mechanisms of Al_2O_3 atomic layer deposition on synthetic polycrystalline MoS_2. *J. Chem. Phys.* **146**, 052810 (2017).

21 Vervuurt, R. H. J., Kessels, W. M. M. E. & Bol, A. A. Atomic layer deposition for graphene device integration. *Adv. Mater. Interfaces* **4** (2017).

22 Nam, T., Seo, S. & Kim, H. Atomic layer deposition of a uniform thin film on two-dimensional transition metal dichalcogenides. *J. Vac. Sci. Technol. A* **38**, 030803 (2020).

23 Lee, H.-B.-R., Baeck, S. H., Jaramillo, T. F. & Bent, S. F. Growth of Pt nanowires by atomic layer deposition on highly ordered pyrolytic graphite. *Nano Lett.* **13**, 457-463 (2013).

24 Oh, I.-K., Sandoval, T. E., Liu, T.-L., Richey, N. E. & Bent, S. F. Role of precursor choice on area-selective atomic layer deposition. *Chem. Mater.* **33**, 3926-3935 (2021).

25 Khan, R. *et al.* Area-selective atomic layer deposition using Si precursors as inhibitors. *Chem. Mater.* **30**, 7603-7610 (2018).

26 Wojtecki, R. *et al.* Additive lithography-organic monolayer patterning coupled with an area-selective deposition. *ACS Appl. Mater. Interfaces* **13**, 9081-9090 (2021).

27 Pasquali, M. *et al.* Understanding selectivity loss mechanisms in selective material deposition by area deactivation on 10 nm Cu/SiO_2 patterns. *ACS Appl. Electron. Mater.* **4**, 1703-1714 (2022).

28 Saha, J. K., Ahn, Y., Kim, H., Schatz, G. C. & Jang, J. Small size limit to self-assembled monolayer formation on gold(111). *J. Phys. Chem. C* **115**, 13193-13199 (2011).

29 Zhang, Z., Ahn, Y. & Jang, J. Molecular dynamics simulations of nanoscale engravings on an alkanethiol monolayer. *RSC Adv.* **7**, 35537-35542 (2017).

30 Saha, J. K., Kim, H. & Jang, J. Nanometer-wide lines of self-assembled monolayer: a molecular dynamics simulation study. *J. Phys. Chem. C* **116**, 25928-25933 (2012).

31 Weckman, T. & Laasonen, K First principles study of the atomic layer deposition of alumina by TMA–H2O-process. *Phys. Chem. Chem. Phys.* **17**, 17322-17334 (2015)

32 Andersen, M., Panosetti, C. & Reuter, K. A practical guide to surface kinetic Monte Carlo simulations. *Front. Chem.* **7**, 434159 (2019).

33 Haseltine, E. L. & Rawlings, J. B. Approximate simulation of coupled fast and slow reactions for stochastic chemical kinetics. *J. Chem. Phys.* **117**, 6959–6969 (2002).

34 Chatterjee, A. & Voter, A. F. Accurate acceleration of kinetic Monte Carlo simulations through the modification of rate constants. *J. Chem. Phys.* **132**, (2010).
